# The experience of closeness and distance in the therapeutic relationship of patients with different attachment classifications: an exploration of prototypical cases

**DOI:** 10.3389/fpsyt.2023.1029783

**Published:** 2023-06-15

**Authors:** Sharon Egozi, Alessandro Talia, Hadas Wiseman, Orya Tishby

**Affiliations:** ^1^Research Center for Innovation in Social Work (RCISW), Tel-Hai College, Tel Hai, Israel; ^2^Primary Care Unit, Department of Public Health and Primary Care, University of Cambridge, Cambridge, United Kingdom; ^3^Department of Counseling and Human Development, University of Haifa, Haifa, Israel; ^4^Department of Psychology, Hebrew University, Jerusalem, Israel

**Keywords:** psychodynamic therapy, attachment style, therapeutic relationship, therapeutic alliance, therapeutic-distance, relationship narratives, relationship-anecdote-paradigm, client characteristics

## Abstract

**Background:**

Individuals with different attachment classifications (Secure, Avoidant and Preoccupied) may experience emotional closeness differently, in their intimate relationships but also as clients in psychotherapy. However, evidence for this assumption almost exclusively comes from research with self-report questionnaires.

**Aims:**

In this paper, we use observer-rated measures to explore in depth how patients with different attachment classifications experience closeness and distance from the therapist in different phases of therapy.

**Method:**

Three patients’ and their therapists’ narratives about the therapeutic relationship at three time points during therapy were extracted and analyzed with two transcript-based observational measures: The Patient Attachment Coding System (PACS), which classifies patients’ attachment according to their discourse behavior, and the therapeutic-Distance Scale-Observer version (TDS-O), which assesses the therapeutic relationship in terms of closeness, distance, autonomy and engagement. Cases were chosen from a larger research project due to their different prototypical attachment classification on the PACS. The narratives were obtained from Relationship Anecdote Paradigm (RAP) interviews in which the patients and their therapists narrated separately about meaningful interactions with each other, at early, middle and late phases of therapy. In addition, we followed patients self-report of the alliance and symptoms (OQ-45).

**Results:**

Although all patients reported experiencing discomfort with feeling distant from the therapist the therapeutic distance, the secure patient was able to reflect on his feelings and, in the therapist’s recollection, was able to share them with the therapist. This allowed the therapist to harness these feelings for the benefit of the therapy. The avoidant and the preoccupied patients both experienced the therapist as distant, but the avoidant patient prevented closeness by a minimal expression of feelings, and the preoccupied described strong frustration with the therapist in a one-sided manner that prevented collaborative processing and left the therapist confused.

**Discussion:**

It appears that patient discourse is a stable (trait-like) component of attachment, while the therapeutic-distance is a process (state-like) component that may change along therapy. The discourse of insecure patients may hinder therapists’ ability to adjust the therapeutic-distance to patients’ needs. Therapists’ knowledge about the ways patients with different attachment classifications communicate their proximity wishes may improve their attunement.

## 1. Introduction

The relationship between patient and psychotherapist is considered a key component of psychotherapy, capable of bringing about change (1–3). Research has shown that the quality of the therapeutic relationship contributes to differences in therapy outcome to a significant degree (1, 4); yet, our understanding of the mechanisms through which the therapeutic relationship can influence change remains incomplete and in search of sharper definition.

One of the most popular conceptualizations of the patient–therapist relationship in psychotherapy is the one first proposed by Bowlby (5) in his attachment theory. Bowlby proposed that the patient–therapist relationship could be considered an attachment relationship, given that it is one in which the patient seeks protection and support from the therapist. Bowlby further hypothesized that the therapeutic relationship would evoke in patients their generalized representations of attachment relationships, which had emerged on the basis of early attachment experiences with their parents, and would orient individuals’ behavior in close relationships. For this reason, Bowlby hypothesized that patients with less felicitous attachment experiences could face difficulties in establishing connection and emotional intimacy with the therapist.

Nevertheless, through the experience of a different, more supportive relationship, Bowlby thought that patients could be helped to revise their Attachment models and achieve greater interpersonal security. After Bowlby, other scholars have elaborated on the process of building stronger patient–therapist attachments and the conditions that allow the therapist to become an attachment figure for the patient (6, 7).

The attachment-informed study of the therapeutic relationship has attracted considerable attention (8–10), but still has significant limitations. In particular, such studies are often conducted through self-report questionnaires, such as the widespread Experiences in Close Relationships Scale [ECR; (11)], or specifically in relation to the therapist through the Patient Attachment to Therapist Scale [CATS; (12)]. For a review of the most commonly used attachment measures in psychotherapy research, see Levy and Johnson (13) and Strauss et al. (14). In addition to their known limitations (15), self-report measures may only summon the patient’s conscious expectations about intimacy, whereas Bowlby had hypothesized that attachment-related differences would not be accessible to consciousness (14). For this reason, attachment-oriented psychotherapy researchers have suggested the need to develop appropriate measures that go beyond the self-report perspective (16, 17).

In this article, we explore how patients with different attachment classifications experience closeness and distance in the therapeutic relationship at different phases of therapy by using prototypical clinical examples. To do so, we conducted a preliminary study employing two attachment-informed observational measures for assessing the therapeutic relationship: the Patient Attachment Coding System [PACS; (18, 19)], and the Therapeutic Distance Scale–Observer version [TDS-O; (20, 21)]. The PACS is an established transcript-based instrument that analyses patients’ discourse behavior and classifies their attachment patterns according to how discourse influences patient–therapist emotional proximity at an observed level. The TDS-O, developed more recently, is a transcript-based measure designed to assess therapeutic relationships in terms of patients’ expressed needs for closeness-distance, autonomy, and engagement in psychotherapy (22, 23).

### 1.1. Attachment in adults

Bowlby posited that individuals develop mental representations of self and others based on early attachment experiences with their caregivers. He called these representations *internal working models* [IWMs; (24–27)]. According to attachment theory, IWMs play a central role as mechanisms of continuity between early experiences with caregivers and later socio-emotional development since they help to anticipate, interpret, and guide interactions with friends, romantic partners, children, and therapists (28–30). Attachment research in early childhood has relied on Mary Ainsworth’s pioneering work using the Strange Situation to classify infants into three attachment types: secure, avoidant, and ambivalent. Mary Main later developed the Adult Attachment Interview [AAI; (31, 32)] to assess attachment-related differences in adulthood. Based on individuals’ narratives of their experiences with caregivers, the attachment representations were classified into three attachment types corresponding to the infant classifications: secure, dismissing, and preoccupied.

Another model describing attachment in adults was formulated by Griffin and Bartholomew (33). They described individual differences in IWMs by locating the individual in two-dimensional space defined by attachment avoidance and attachment anxiety. This representation reflects both the person’s sense of attachment security and the ways in which they deal with threats and distress. Individuals who score low on these dimensions are generally secure and tend to employ constructive and effective affect-regulation strategies and interpersonal contacts. Those who score high on either the anxiety or the avoidant dimension (or both) suffer from attachment insecurities and tend to rely on secondary attachment strategies (34–36).

Individuals who are high in attachment anxiety and low in attachment avoidance rely on hyperactivating strategies. They intensify dependency needs and wish for closeness in their relations with attachment figures. These people tend to seek greater proximity to the attachment figure, exhibit maximal amplification of attachment behaviors, and show hypersensitivity to any sign of rejection. Individuals who are low in attachment anxiety and high in attachment avoidance rely on deactivating strategies. They increase distance so as not to get hurt (37) and tend to divert their attention from distressing situations or from attachment-related thoughts and emotions (36). Both of these secondary attachment strategies might form a challenge for therapists who aspire to find ways to work through patients’ insecurities and establish a collaborative alliance (29, 38).

### 1.2. Attachment in psychotherapy

Pre-treatment attachment differences have been examined in psychotherapy research both as a predictor of outcome and as a moderator of change. A recent meta-analysis by Levy et al. (39) demonstrated that patients with secure attachment pre-treatment showed better psychotherapy outcomes than patients with insecure attachment. Further, it revealed that improvements in attachment security during therapy (i.e., with the therapist) may coincide with better treatment outcome [e.g., (8)]. Finally, Levy et al.’s preliminary moderator analyses suggested that those who experience low pretreatment attachment security may find better treatment outcome in therapy that incorporates a focus on interpersonal interactions and close relationships. This finding may point to the need for therapists to find ways to enhance secure therapeutic attachment, especially among insecure patients.

Despite the understanding we have gained about the relationship between patients’ attachment and therapy outcome, much less is known about how change in attachment occurs. More research is needed to understand how therapeutic attachments are formed and develop during therapy, especially with insecurely attached patients, and how patients’ internal working models may affect therapists’ responsiveness.

Mallinckrodt and his colleagues addressed this question (22, 23, 29). They introduced the concept of *therapeutic distance*, which they defined as “the level of transparency and disclosure in the therapeutic relationship from both patient and therapist, together with the immediacy, intimacy, and emotional intensity of a session” [(22), p. 559]. They suggested that expert therapists regulate the therapeutic distance according to patients’ attachment needs and phase of therapy.

Thus, since hyperactivating patients tend to feel that their therapist is too distant (e.g., cold, remote, or not helpful enough), Mallinckrodt suggested that at the beginning of therapy with these patients, therapists should attempt to minimize the therapeutic distance in order to allow the patient to feel safer. As therapy progresses, therapists should then strive to gradually increase the therapeutic distance in a manner that allows their patients to experience autonomy and independence, for example by encouraging their self-reliance and by supporting their independent decisions.

In contrast, they proposed that deactivating patients tend to perceive their therapist as too close (e.g., pushing for disclosure and emotional proximity to an excessive degree). Therefore, they suggested that during the initial phase of therapy, therapists should respect these needs by allowing a measure of therapeutic distance. Only during more advanced phases should therapists begin to challenge these needs for distance by establishing a closer, more engaged and caring relationship (22, 23, 29). In order to test their model, Mallinckrodt et al. (23) developed the Therapeutic Distance Scale (TDS), a self-report questionnaire that measures patients’ experience of therapeutic distance and their feelings of growing autonomy and engagement. The TDS comprises four subscales: *too distant* and *too close* (both referring to patients’ experience of therapeutic distance), and *growing autonomy* and *growing engagement* (both referring to the expansion of their IWMs). A preliminary study provided initial support for the TDS construct validation (23).

### 1.3. Patient Attachment Coding System

The Patient Attachment Coding System (PACS) also assesses attachment in therapeutic relations. It is based on the assumption that people implicitly use their discourse to regulate closeness and connection with the other, including the therapist (18, 19). Using psychotherapy session transcripts, the authors found that various patterns of in-session communication reliably predicted differences in patients independently obtained by Adult Attachment Interview classifications [AAI; (31)].

In the PACS, secure patients convey their present experience openly. They disclose their emotions in the here-and-now and share vivid narratives of past experiences that clearly convey their current feelings. Secure patients also communicate needs in the therapeutic relationship and share their present intentions, autonomous reflections, and positive experiences. These speech acts, rated on the PACS scales *proximity seeking* (distressful emotions in the here-and-now), *contact maintaining* (positive feelings like appreciation, gratitude and love toward the therapist or therapeutic process), and *exploring* (examining internal states in the “here-and-now”) allow the therapist to take part in the patient’s experience, reflect or elaborate by asking questions, and increase closeness.

Avoidant patients tend to decline requests to express their feelings or are reluctant to describe their experiences in sufficient detail; they tend to downplay recent emotional experiences (positive or negative) and convey unwillingness to change. These types of communication, rated by the PACS *avoidance scales*, preempt any offer of support and connection by shifting the listener’s attention away from the speaker’s internal state. Preoccupied (anxious) patients share their experience in a one-sided, exaggerated, or confusing way that leaves little room for the therapist to respond. For example, they may persuade the therapist to join their point of view (*involving* markers), or convey their experience in an impersonal, difficult to understand way (*merging* markers). These patterns tend to limit the extent to which the therapist can make meaning of patients’ experience, leaving no room for contradiction, challenge, or elaboration, and they actually disregard the therapist’s interventions.

Studies using the PACS with different patient samples in a range of therapeutic modalities have confirmed that AAI classifications predict marked differences in patients’ in-session communication, and that by analyzing such differences in a single session, one can predict patients’ AAI classifications (19). One study also identified these communication patterns in post-treatment interviews conducted by an unfamiliar interviewer, not a psychotherapist (40, 41). Moreover, the PACS *exploring* scale has been shown to predict patients’ ratings of mentalizing assessed independently (40, 41); PACS *security* scales have also predicted greater resolution of alliance ruptures (42, 43) and greater physiological synchronization between patient and therapist (44).

### 1.4. The therapeutic distance scale–observer version (TDS-O)

Following Mallinckrodt and Jeong’s (45) model, and taking a relational perspective (46), the TDS-O was developed to track the unique dyadic dance between patient and therapist, focusing on the closeness–distance experiences of both patients and therapists. The TDS-O expanded the TDS by creating an observer-narrative-based version [TDS-O; (20, 21, 47)], which enables the researcher to follow both the patient’s and the therapist’s experiences through their descriptions of their interactions with each other. The narratives told by patients and therapists provide a window into subjective significant moments at different phases of therapy by coding the narratives on the four scales of the TDS-O. While coding by judges entails interpretive aspects, at the same time they also allow identifying the implicit needs, expectations, and feelings of each of the partners, which may not be accessible by means of self-report questionnaires. In a previous study, Egozi et al. (20), showed that patients’ and therapists’ experiences of therapeutic distance change during the course of therapy. Patients showed a decrease in perceiving therapists as *too distant* and an increase in engagement, and therapists showed a decrease in perceiving patients as *too close* and an increase in granting *autonomy* and *engagement*.

Convergent validity of the TDS-O was established through associations with self-report measures—the attachment measure of the ECR and the Working Alliance Inventory [WAI; (48)]. In these studies, patient and therapist narratives were analyzed by trained observers using the TDS-O at three phases of therapy. During the same phases, the patients and therapists answered the self-report questionnaires. The first study (21) showed that attachment characteristics shaped the closeness–distance dynamics. Appling a dyadic approach to examine associations between attachment patterns of patients and therapists, as measured by the ECR, and their experience of therapeutic distance, the study showed that patient *attachment anxiety* related to different proximity needs than patient *avoidance*, and both needs varied along therapy. Therapist *anxiety* motivated more closeness with patients but was not always congruent with patient experience. Therapist *avoidance* impeded attaining optimal distance in the therapeutic dyad, especially during the initial phase. The TDS-O enables us to follow congruence and incongruence between patient and therapist experiences regarding their proximity, and thus has the potential to contribute to therapist ability to attune to patient emotional needs at the specific moment (49). The second study (47), demonstrated significant relations between the TDS-O and the therapeutic alliance. Patient and therapist decrease in their sense that the partner was *too distant* correlated with increase in the alliance, as did therapist decrease in the sense that the patient was *too close*. Increases in both partners’ engagement were also related to alliance improvement.

Overall, the TDS-O appears to be a promising attachment-informed measure that allows following change in the therapeutic relationship between patient and therapist along therapy from the perspectives of both patients and therapists.

Following Strauss et al. (14), who pointed to the need to define and interpret the convergence and divergence of different attachment measures, we suggest that although both the PACS and the TDS-O are attachment-informed, each focuses on different aspects of attachment: The PACS classifies patients by attachment patterns (*secure*, *avoidant*, or *preoccupied*) and the TDS-O follows the closeness–distance dynamics along therapy.

### 1.5. The present study

This article presents a preliminary study designed to explore the various pathways that patients with different attachment classifications, according to the PACS, experience. It relies on measures of *closeness*, *distance*, *engagement* and *autonomy* (according to the TDS-O) at different phases of therapy, using three prototypical clinical cases. We also analyzed the therapists’ narratives in order to evaluate how the therapist understands the patient’s closeness–distance needs, and possible effects of the patient discourse pattern on these perceptions. To enrich our understanding of the patient–therapist encounter and outcome we also examined patients’ self-report of the therapeutic alliance, as well as their symptom change.

## 2. Method

### 2.1. Patients

The cases for this study were selected from a larger research project at a university counseling center (50, 51). In this study, the patients were 67 young adults suffering from distress for which they sought treatment at the community psychological services provided by the university they were attending. Most of them were diagnosed with either mild depression and/or anxiety, presenting with difficulties in relationships, in academic studies, or issues pertaining to the formation of their identity. Twenty-five patients (17 females and eight males) were randomly selected from the larger sample recruited for the project, and their interviews were classified with the PACS. Of these 25 patients, seven were classified as secure, seven as avoidant, and 11 as preoccupied.

For this paper, we selected three patients who most clearly represented the three prototypical attachment communication patterns classified on the PACS (secure, avoidant, and preoccupied), namely, those who received the highest prototypical values specific to the respective attachment classifications as agreed by two independent judges (the first and second author). Although this was not a selection criterion, the three patients started therapy with a symptom level higher than the clinical cut-off (> 63 according to the OQ–45, see below).

### 2.2. Therapists

Twenty-nine therapists participated in the large research project. They held MA degrees in clinical psychology or clinical social work. The therapists of the three prototypical patients in this study were all females, aged 32–34, who were at the advanced stage of their internship at the university counseling center.

### 2.3. Therapy

Treatment was provided at the university psychological services of a large university. The therapeutic approach of the therapists was psychodynamic, based on the core principles outlined by Summers and Barber (52), which were employed in all therapies. All the therapists received weekly individual psychodynamic supervision. Treatment consisted of weekly 50-min sessions and was not defined *a priori* as time limited. Median treatment length was 14 months. The therapy is not protocol-based and reflects psychotherapy practice in Israel. [For further details on the sample and the therapy, see (51)].

### 2.4. Measures

#### 2.4.1. Relational anecdote paradigm (RAP) interviews

All patient and therapist participants were administered the Relational Anecdote Paradigm (RAP) interview (53). In this interview, participants were asked to choose and describe three meaningful interactions that had recently taken place with their partner in the therapeutic dyad (either the therapist or the patient). The interviewer asked the participant about their feelings and thoughts during the interaction [for details see (17)]. Patients and therapists were interviewed separately by different interviewers without being exposed to their partner’s narratives. The interviews were held three times over the course of therapy: early phase—after session 5, mid phase—after session 15, and late phase—after session 28. Session 5 was chosen order to assess the emerging alliance, sessions 15 and 28 were chosen as time points that reflect deep therapeutic work. Session 28 was also close to the end of the academic year, and either preceded termination or a short break before the second year. Thus, we wanted the measurements to reflect a continuous therapy process uncolored by termination or separation.

In total, each patient and therapist related nine narratives (three per time point) about their therapist or patient, respectively. The interviews were conducted during the week following the designated session, depending on scheduling constraints (some the following day and up to a few days later). The PACS and TDS-O were applied independently to the nine narratives of each patient.

#### 2.4.2. Attachment-informed observer narrative-based measures

##### 2.4.2.1. Patient Attachment Coding System

The Patient Attachment Coding System [PACS; (18, 40)] assesses patients’ attachment classification by examining their verbal communications in a clinical setting with a therapist or an interviewer. The measure can be applied to any transcribed therapy session or interview, regardless of the therapy type or the content of the topics discussed. The PACS was developed following the identification of a number of in-session discourse characteristics that were shown to be statistically associated with patients’ independently obtained AAI classifications (18, 40). The occurrence of these characteristics in a psychotherapy session transcript leads to the assignment of one of three PACS attachment classifications (secure, avoidant, or preoccupied). To code with the PACS, the transcript is rated as a whole, without segmenting the text. The coder first identifies in the transcript any number of the 59 discourse markers described in the coding manual. The markers refer to distinct ways of communicating about present internal experience. Each marker belongs to one of five main scales, which are rated from 1 to 7, based on the frequency and intensity of the markers.

*Proximity seeking* rates the disclosure of painful emotions and narratives about distressing experiences; *contact maintaining* rates communications about the positive impact of the therapy or the therapist; *exploring* rates in-the-moment reflections (occurring in present time) about mental states, expressions of self-assertion and intentions, and reports of positive experiences; *avoidance* rates instances in which the patient fails to disclose in response to the conversation-partner’s queries (subscale: *direct resistance*) or downplays the magnitude or importance of their experience (subscale: *releasing*); *resistance* rates instances in which the patient ignores or changes topic in response to the conversation-partner’s queries (subscale: *direct resistance*), or communicates about internal experience in excessive, vague detail, or in exaggerated, one-sided terms (*involving* or *merging* subscales).

The PACS classification is obtained using an algorithm based on the scores of the five main scales. Secure patients show high ratings on the *proximity seeking*, *contact maintaining*, and *exploring* scales and low ratings on *avoidance* and *resistance*, while avoidant and preoccupied patients show high ratings on *avoidance* and *resistance* respectively, and low ratings on the scales associated with security. The authors reported good concurrent validity with the Adult Attachment Interview [AAI; (31)]. For details on PACS psychometric properties and more detailed descriptions of the markers and scales, see Talia et al. (19).

###### 2.4.2.1.1. PACS rating

To implement the PACS on patient’s RAP interviews, the first author was trained by the author of the PACS, Talia, to use the PACS and reached sufficient reliability when rating therapy-session transcriptions with him. At the next phase, for the purpose of establishing inter-judge reliability, 14 of the current study’s RAP interviews (56%) were translated from Hebrew into English and rated by both Talia and the first author. They obtained 86% agreement on the global PACS classifications.

The three patients selected for the current study were rated by the first and second authors and there was an agreement on their classifications. They were chosen as prototypical cases of the three PACS classifications: *secure*, *avoidant*, and *preoccupied* (see the limitations section).

##### 2.4.2.2. Therapeutic distance scale-observer version (TDS-O)

The TDS-O (20, 21) is based on the self-report measure developed by Mallinckrodt [TDS; (23)]. For the observer version, the items of the original questionnaire were adapted to assess the narratives related by the patients and the therapists about each other in their RAP interviews. Thus, two versions were constructed—one to assess patient narratives and the other to assess therapist narratives. In the patient version, the focus of the four scales was on their own experiences. In the therapist version, the *too distant* and *too close* scales focused on the therapists’ own experiences, and the *autonomy* and *engagement* scales dealt with their attempts to enable these components for their patients. The following is a description of each scale and relates to the two versions:

*Too Distant*: A 7-item scale that measures the extent to which the partner is perceived as distant and inaccessible. In the patient version, the emphasis is on the extent to which the patient perceives their therapist as cold, distant, and unhelpful, for example, “There are times when the counselor seems cold and personally distant”; in the therapist version, the emphasis is on the extent to which the therapist feels rejected by their patient and unable to assist them, for example, “Therapist feels that he/she has not been helpful to the patient.” *Too Close*: A 7-item scale that measures the extent to which the partner is perceived as intrusive and forcing closeness. For example, in the patient version, “The patient feels that the therapist wants them to reveal too much personal information”; in the therapist version, “The patient insists on pursuing a topic even though the therapist does not want to go there.” *Autonomy*: A 6-item scale that measures the extent to which therapy empowers the patient, encouraging them to make independent decisions and take the initiative. For example, in the patient version, “The therapist helps the patient generate their own solutions instead of telling them what to do”; in the therapist version, “The therapist feels that they are helping the patient to generate their own solutions instead of telling them what to do.” *Engagement:* A 6-item scale that examines the extent to which the therapeutic relationship allows the patient to discuss sensitive issues and relinquish their concerns regarding the need to reveal themselves. The scale emphasizes the changes in the patient’s automatic patterns (distrust and inability to get close) that is unique to their relationship with the therapist. For example, in the patient version, “The patient feels that the counselor has helped them feel more relaxed and comfortable to talk about very personal topics”; in the therapist version, “The therapist feels that they have helped the patient feel more relaxed and comfortable to talk about very personal topics.”

###### 2.4.2.2.1. Scoring the TDS-O

The raters were asked to rate each item on a 6-point Likert scale from 1 (*not at all present*) to 6 (*strongly present*). They were instructed to first read the narrative transcript as a whole, and then rate the extent to which each item appeared in the narrative. The three narratives in each of the three interviews during psychotherapy were rated separately, so that a total of nine assessments were received for each participant: three narratives at three different time points (18 narratives for each dyad). Then, we calculated a mean interviewee score across the three narratives of each time point, so that each participant had a total of 12 different scores: four scores for each of the TDS-O scales for the three time points. The mean scores, like the scale, ranged from 1 to 6, so that high scores represented a high degree of presence in the narrative. Inter-rater reliability of the TDS-O items (α Cronbach) ranged from 0.86 to 0.97. For further details on the rating process, including internal reliability and validity of the TDS-O, see Egozi et al. (21).

#### 2.4.3. Self-report measures

##### 2.4.3.1. Working Alliance inventory

The WAI (48) is a widely used 36-item self-report questionnaire that was developed based on Bordin’s (54) conceptualization of the alliance; it consists of three subscales: *bond*, *task*, and *goal*. Each item is rated on a 7-point Likert scale from 1 (*lowest alliance rating*) to 7 (*highest alliance rating*). The psychometric properties of the WAI are well-established (55). A Hebrew version of the scale yielded high internal consistency, in the present study α = 0.87. The patients answered the questionnaire three times: at early phase (session 5), mid phase (session 15), and late phase (session 28).

##### 2.4.3.2. Outcome questionnaire (OQ-45)

The OQ-45 (56), is a self-report instrument designed for repeated measurement of client symptom changes throughout therapy. Clients are asked to rate their functioning during the past week on a 5-point Likert scale, from 0 (*never*) to 4 (*almost always*), five times along therapy: at intake, early phase (session 5), mid phase (session 15), late phase (session 28), and follow-up (after session 32). The OQ-45 consists of symptom distress, interpersonal problems, and social role. It has adequate test–retest reliability (0.84) and high internal consistency (0.93). Concurrent validity has been demonstrated with a wide variety of self-report scales (e.g., Beck Depression Inventory, State–Trait Anxiety Inventory). Using formulas developed by Jacobson and Truax (57), the reliable change index of the OQ has been estimated at 14 points (56); that is, participants whose change was in a positive or negative direction by at least 14 points are regarded as having made reliable change. The OQ-45 is widely used and has been translated into several languages, including Hebrew (58). The clinical cut-off score in the Israeli sample was 63, similar to the cut-off score in the U.S., and the reliable change index was also the same. In the present study, the alpha coefficient of the total OQ-45 was 0.91 (50).

## 3. Prototypical cases: the meeting of the lenses of the PACS and TDS-O

To illustrate how patients with different attachment classifications (according to the PACS) experience the therapeutic distance in their relationship with the therapist at different phases of therapy (according to the TDS-O), we describe a detailed analysis of the narratives of three patients according to the two measures. For each case, we use examples from the RAP interviews, presenting the characteristics of the therapeutic distance and how they developed through therapy, as well as the discourse patterns as reflected through the PACS rating. In addition, we examine the therapists’ narratives to understand how they experienced the relationship, and the possible impact of the patient’s discourse patterns on the therapist’s experience of therapeutic distance with their patient. We also provide the scores on the self-reported alliance (WAI) and the outcome from the patient’s point of view (OQ-45) for each case. It should be noted that these scores were not taken into consideration in the choice of these cases. Instead, the WAI and OQ-45 scores were drawn from the larger study in retrospect in order to provide self-report information on the alliance of the patients over time, as well as OQ-45 scores pre-and post-therapy. These measurements are used to discuss the added value obtained from using the observational attachment-informed indices.

### 3.1. Secure patient: Doron

**Figure 1:**
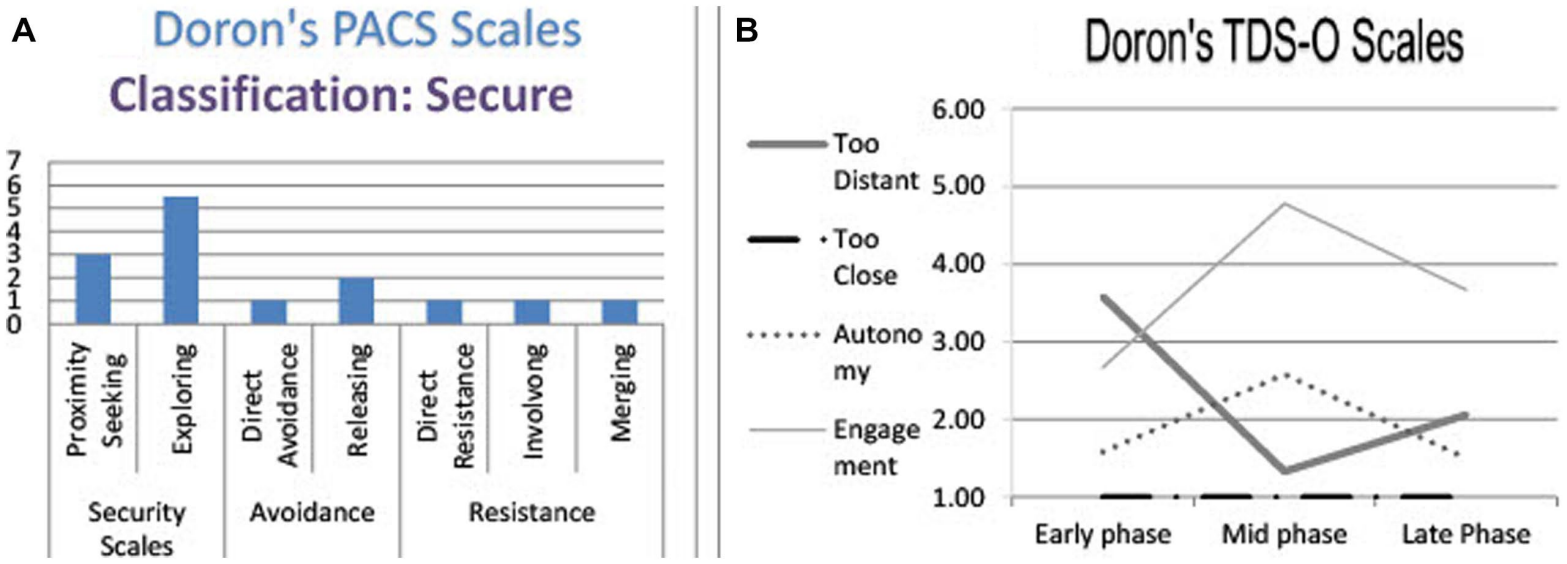
Doron’s TDS-O and PACS markers. **(A)** Doron’s PACS scales. **(B)** Doron’s TDS-O scales.

Doron is a single, 24-year-old male undergraduate student. He was classified secure on the PACS: During all three interviews he exhibited a great many *exploring* and *proximity seeking* markers (see Figure 1A). The TDS-O graph shows a moderate decrease in his feeling that the therapist was too distant and an increase in engagement at the middle phase, as well as some decrease in engagement and some increase in too distant at the late phase (Figure 1B).

*Working Alliance and OQ self-report measures:* Doron’s working alliance scores were relatively high and stable along the three measurements (5.36, 5.19 and 5.28 respectively). His OQ scores decreased from 66 at intake (above clinical cut-off) to 45 at the follow-up (reliable change). The OQ scores seem relatively stable in the early and middle phases (66 and 65 respectively) and then decreased to 54 in the late phase and continued to show improvement at follow-up.

#### 3.1.1. Early phase (after session 5)

##### 3.1.1.1. Patient narrative

During the interview, Doron described a desire for the therapist to get to know him, see his uniqueness, and learn to like him. The narratives contain attempts to impress the therapist, but Doron discussed complex emotions that arose in this phase:

*I have this persona that I enjoy playing, of an English gentleman, and it protects me … keeps me away … I started playing this character and it really amused her. I like to entertain, and I enjoyed seeing her laugh. But it was complicated—because it felt like I was doing exactly what I came to therapy not to do… I think maybe I expected her to understand that it was something I had to show her, but not to take too seriously. Maybe she understood that this is the game of my life, at home, in my dealings with myself.*

In the second narrative, he described another attempt to initiate a deeper mutual connection:

*I was preoccupied with something before the session and read a passage I had written about it in my notebook. Then, when I went into the session … I read this passage to her. It was my way of testing her: Here, I share with you my own inner discourse, with all sorts of fantasies … as if I was checking how much you could understand me, even when I am in such a distant place. But when reading it to her, I censored one line—something I wrote about love, something more sexual that I felt I wouldn’t like to reveal to her, not yet. I think it was a showoff situation—I introduced her to my qualities: my theatrical skills and my ability to write. Like, this is part of who I am, something I ask you to know, to understand, but also to find its beauty and be impressed.*

Both narratives are characterized as exploring according to the **PACS:** Doron described a “defining moment” in a vivid and emotional way, allowing a listener to respond in a variety of fashions (identify, ask questions, elaborate, etc.). He *reflects* on his behavior, trying to explain gaps between opposing perceptions: The need to impress and “show off” and the longing for love; the need to amuse and please; and the desire that the therapist would be able to understand the defenses involved.

In terms of the TDS-O, the narratives are evaluated as *too distant* (medium level). Doron described a longing for closeness, but challenged the distance by initiating interactions that he hoped would allow a better acquaintance. The fact that he initiated the approach and did not leave it in the hands of the therapist resulted in a medium score on the scale. In addition, he ensured that he would not create too much intimacy by maintaining the theatrical “English gentleman” persona in the first narrative, and by censoring the text in the second. In fact, he expressed a hidden fear of the therapist becoming *too close*, but since he did not attribute it to the therapist and considered himself responsible for the degree of closeness between them, he received the minimum rating on the *too close* scale.

##### 3.1.1.2. Therapist’s TDS-O markers

The therapist also felt that they had still not managed to find the right distance. She felt *too distant* (for example, when having difficulties finishing the session on time, and felt guilty for “*having to dump him out*”), as well as *too close* (for example, when offering an interpretation and afraid of being “*too deep too early*”). Nonetheless, she seemed optimistic about their future relationship: “*Maybe there is no good way of saying goodbye now (at the end of the session), but we may be able to talk about it sometime*.”

In summary, during the first interview both patient and therapist felt they had not yet managed to find the right distance, but they both attributed this to their short acquaintance and looked forward to a change for the better. Doron, as a secure patient, showed initiative and opened dialogue about feelings and thoughts, which allowed the listener to participate in his experience and respond in a variety of ways. His relatively high alliance rating at this early phase strengthens this interpretation. Although therapist and patient were interviewed separately, the feelings they described about their closeness–distance dynamics appear quite synchronized.

#### 3.1.2. Mid phase (after session 15)

##### 3.1.2.1. Patient’s narrative

Doron’s narratives were less preoccupied with closeness–distance at this phase. It seems as if he felt their relationship had established the “right distance,” and the narrative revealed more aspects of engagement and autonomy. For example, he related that they decided to discuss his fear of death in the next session, but during that session he began talking about other things:

*I felt I’m not able to start talking about that. I had to speak about other topics first. Not to get into this immediately. I expected her to notice … and I guess she did. She referred to it, but not as if I’m wasting time or anything, but also not as if it’s the “main course.” I felt good. I felt that I’m not forced to speak. It was important for me to be in a place where I can feel emotionally connected to the things I’m talking about, where I feel safer. She understood that, and it made me feel comfortable.*

In terms of the **PACS,** Doron’s discourse is characterized as an *affective sharing*: He praised the therapist’s ability to be attuned to his needs and expressed his gratefulness. The open description of his feelings allowed the listener to identify with him and feel closer.

His **TDS-O** rating was low in both *too distant* and *too close*, since Doron did not seem troubled by their proximity. He implied autonomy—the therapist let him set the rhythm of the conversation. By doing so, he felt understood and encouraged. The therapist allowed him to feel safe and gradually reached the difficult topic (his fear of death) and therefore, Doron rated high on the *engagement* scale.

##### 3.1.2.2. Therapist’s TDS-O markers

At the same time, the therapist described a rupture: She related to a moment in which Doron expressed difficulty connecting to therapy. At first, she felt guilty for not being helpful enough (too distant), but she moved quickly to the “here-and-now”—they understood, together, that the “*difficulty in feeling connected*” was a part of the issues he brought to the room, and they may have to “*learn to bear it for some time, in order to understand it.*” In her intervention, the therapist helped to contain the distance and transformed it from a “separating” to a “connecting” component, in which both shared the expectation of developing their relationship beyond this barrier (therefore, she encouraged engagement). She also granted autonomy by allowing Doron to dictate the pace and conveyed a belief in his ability to move forward.

In summary, during the mid phase, both partners were less occupied with the therapeutic distance, as expected during the working phase. Doron was grateful for the secure base the therapist provided (which can also be seen in his high rating of the alliance). The therapist, although momentarily feeling distant and unhelpful, was able to use this feeling to repair the collaboration and be flexible in the therapeutic tasks.

#### 3.1.3. Late phase (after session 28)

##### 3.1.3.1. Patient’s narrative

During this late phase, Doron’s narratives reflected several aspects of the therapeutic distance: On the one hand, he felt close and comfortable with the therapist, but on the other, there was a desire to approach the therapist a little more (perhaps beyond what is possible in therapy), together with some critical attitude toward the sense of ease that their proximity produced at the expense of achieving the therapeutic goals.

Example 1:

*There was a moment during our conversation when I suddenly leaned back with some new ease, and it had to do with a few things: First, I have the feeling that she knows me well; I can speak to her in my language and she will understand. I enjoyed talking to her during recent sessions because … she was able to communicate in a way that made me feel very comfortable.*

*But there was also something a little disturbing in this ease—this comfortable place scared me a little. We could sit and talk forever, but somewhere this ease preserves a kind of discourse I had hoped to go beyond. I came here to talk and to feel understood, but also to learn a new language. And this ease—it was something that seemed to be on the border between those two things.*

Here, like in previous phases, Doron’s discourse is characterized according to the PACS by *affective sharing* and *exploring*: He observed and analyzed his feelings in the moment. The ability to acknowledge and understand contradicting emotions is unique to secure patients.

He acknowledged comfort regarding the therapeutic distance (low values of *too distant* and *too close*), in addition to the feeling that the therapist understood his special language (high *engagement*). Nevertheless, he pointed to the limitations of this engagement by expressing concern that it may reduce the ability to explore new regions in therapy.

Example 2:

*I was talking about my fears of a long-term, binding relationship, and then she said that when you really experience a serious, obligating relationship, there is something about it that reduces this great fear. And the moment she said this, I remembered that I saw her once near the coffeeshop I work at. I don’t think she saw me, but she was with her husband and son. And during the session, I really wanted to ask her how she feels … How she experiences living with a person you love … and I didn’t ask her because…I don’t know, I guess I felt that “this is not what we’re here for” … But I had this curiosity … sometimes I want to enrich our relationship. As if, to know her better as a person.*

This narrative, like the previous ones, is characterized by PACS *exploring* markers: by describing a defining moment and how it challenged their relationship, Doron would have allowed the listener to enter his inner world.

In terms of therapeutic distance, Doron sensed that the therapist was a bit more distant than he would have liked her to be. He wanted to know more about the therapist as a person, but unlike in the first interview, he did not initiate an approach, due to his understanding of the boundaries of the therapeutic relationship. He acknowledged a certain sense of sadness, together with an acceptance that this, probably, cannot be changed.

##### 3.1.3.2. Therapist’s narrative

At late therapy, the therapist emphasized her ability to respond freely to Doron:

*With him I can just talk about it. I told him that I have this feeling that he is not connected, and that I can’t reach him. I have the expectation that he will be able to hear it and to accept it. I have other patients I wouldn’t tell that to. And with him—yes. I have the expectation that he can relate to this.*

The therapist felt that she had the space to act in a variety of ways with Doron—she could raise subjects that had the potential to create ruptures. By saying, “*He will be able to hear it and to accept it*,” she implied that she was not concerned about resistance, and that she believed that his ability to explore would allow them to harness these moments for the benefit of the therapy.

In summary, analysis of Doron’s PACS communication patterns, which were prototyped to secure patients, shows an open discourse about negative and positive feelings and the ability to explore gaps in the experience. This seemed to encourage the therapist to continue elaborating on his experience and probably to attune accurately to his relational needs.

The analysis of this prototypical case of a PACS secure patient shows that in terms of the therapeutic distance, the patient allowed a gradual approach and did not leave the responsibility of creating the relationship to the therapist alone. In accordance with the theoretical model (22), preoccupation with the therapeutic distance appears mainly during the early phase of therapy and in the advanced stage (perhaps towards termination?), while in the mid phase the relationship constitutes an infrastructure for the therapeutic work. At the same time, according to the self-report measures, Doron’s alliance rating remained relatively high and stable throughout the three measurements, and his symptom level decreased (good outcome).

### 3.2. Avoidant patient: Rotem

**Figure 2:**
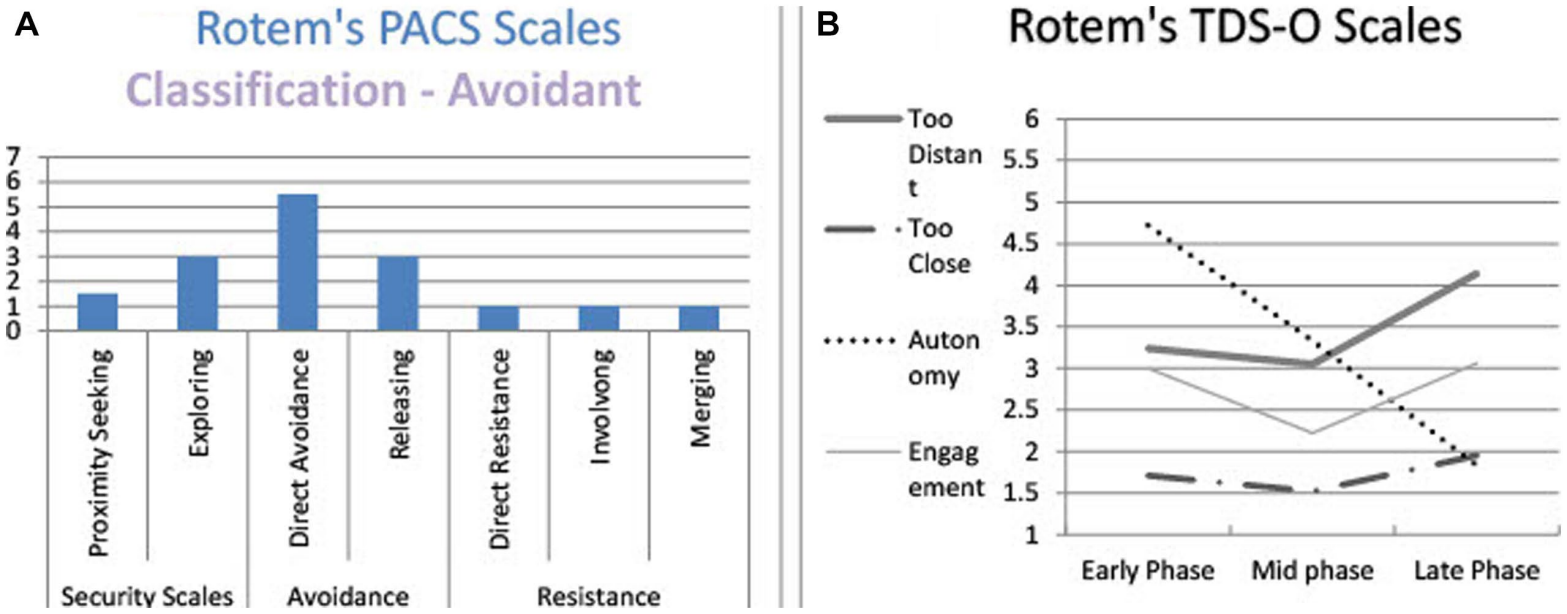
Rotem’s and PACS and TDS-O markers. **(A)** Rotem’s PACS scales. **(B)** Rotem’s TDS-O scales.

Rotem is a 24-year-old female, single, undergraduate student who was classified as avoidant on the PACS. Her narratives contained frequent instances of direct *avoidance* (avoiding direct descriptions of her feelings) and *releasing* markers (shifting the listener’s attention from her mental state). In the TDS-O graph (Figure 2A) we can see a minor increase at the late phase in the feeling that the therapist was *too distant* and a decrease throughout in her *autonomy*. Her narratives do not reveal feeling that the therapist was *too close* at the initial phase (as expected with avoidant patients in Mallinckrodt’s theoretical model), nor an increase in her engagement along therapy. On the contrary, the therapist was perceived as *too distant* at all phases (Figure 2B). In general, the range of emotions arising from Rotem’s narratives was limited. The therapist experienced Rotem as *too distant* along the entire process.

*Working Alliance and OQ self-report measures:* Rotem’s working alliance scores show a medium level at the early phase (4.61) with a decrease with time from mid therapy to late therapy (4.17 and 4.03 respectively). Her OQ scores increased from intake to the early and mid phases (79 to 93 and 95 respectively) showing heightened distress as she attempted to engage in therapy. Then there was a decrease, with a return to close to the intake point (76 and 78, for late and follow-up respectively), but she still remained above the clinical cut-off.

#### 3.2.1. Early phase (after session 5)

##### 3.2.1.1. Patient’s narrative

*I remember that during the first session I didn’t know how to behave … what to talk about. There were moments when I was silent. I did not have much to say. Such confusion and perhaps embarrassment created between two people. I remember that I did not know what to do, how to continue. Perhaps if the therapist had asked specific questions; but on the other hand, I don’t know if it’s in the nature of therapy to ask questions … I guess this is a matter of how to open up…I’m not used to disclosing myself.*

According to the PACS, Rotem’s discourse contains *downplaying:* Immediately after what may be construed as a complaint or request for help, she shifted the interviewer’s attention from her discomfort by claiming that this was probably not the therapist’s fault: “*I do not know if it’s the nature of therapy to ask questions*.”

In terms of the TDS-O, Rotem seemed to perceive the therapist as both *too distant* and *too close*: The therapist did not help her start the conversation (*too distant*), but she was not sure the therapist was supposed to do so (minimized her disappointment), therefore—her rating on the scale was medium-low. The need to be in the same room with the therapist and to disclose was strange and embarrassing (*too close*), but unlike the secure patient, she did not acknowledge her feelings and the observer (and probably the therapist, too) had to interpret them from what little she said about them. Therefore, the values of the *too close* scale were medium-low. Interestingly, at this early phase her symptoms increased, perhaps due to the difficulty caused by the need to open up and get closer.

##### 3.2.1.2. Therapist’s narrative

The therapist related that she had to cancel one of their sessions at the last moment and felt guilty about it. Regarding Rotem’s possible response, she says:

*I had this fear that even if she was mad, she wouldn’t show that anger. If she had expressed this, we would have been able to deal with it, but she wouldn’t say …*

Here, the therapist described the difficulty of getting close to the patient’s emotional experience, due to her avoidant speech patterns.

In another narrative, the therapist described Rotem’s tendency to downplay her emotions and the distancing effect it had on her:

*I felt like I couldn’t connect with her. There was something flat, I did not feel any intense emotions there. I thought about patients I had worked with in the past, who I always felt something immensely powerful about. It’s different with her.*

The therapist felt distant. Although she acknowledged Rotem’s tendency to keep others away from her feelings, she felt guilty for not being able to connect.

#### 3.2.2. Mid phase (after session 15)

##### 3.2.2.1. Patient’s narrative

*I told her that I found it very difficult to self-disclose and to trust. And then she asked if this happens here also, in therapy, and I told her yes, it’s very difficult to reveal myself to you—because this is a situation with a stranger. When I started therapy, I thought it would be very easy to treat me, because I analyze everything and I’m very rational. But then I understood it’s not going to be so easy, since I don’t express emotions, and I’m careful not to enter “dark corners.” I said that I’m afraid I won’t be able to do it. And she said that that is something to relate to…but I also don’t think it can be different. I don’t see how it could be different.*

This narrative, like the earlier one, reveals a *downplaying* discourse pattern: After expressing some frustration with the therapeutic relationship, she claimed it could not be different. In doing so, she released both the therapist and herself from responsibility for change.

Although it may seem that during the interview, Rotem was *exploring* the reasons for her emotional detachment, it was not considered an exploring marker according to the PACS because the thinking was done in the past and was over before the interview; the interviewer in fact had no reason to ask her to elaborate. While reflection and exploration are ways to encourage a listener’s attunement, such a report carried out in the past releases the interviewer from the need to react.

In terms of the TDS-O, Rotem described the therapist as *too distant*. She had difficulty in trusting the therapist, who is perceived as a stranger. Rotem realized she prevented the therapist from getting closer, and yet she “*does not think it can be different*.” She also recognized the therapist’s attempts to engage her, but resisted these attempts by claiming that nothing could change.

Rotem’s alliance decreased at this phase, and her OQ was still higher than at the intake level.

##### 3.2.2.2. Therapist’s narrative

*It is very difficult to connect with her. There’s a big gap between what she conveys and her ability to become intimate. I felt uncomfortable, uneasy…that I can’t really be there for her since she has this laconic speech. She made me feel like “her not-good-enough mother,” as if I was constantly being tested.*

Here too, the therapist felt guilty for being too distant and described her difficulties in feeling empathic and connected to Rotem, due to her “flat” discourse.

#### 3.2.3. Late phase (after session 28)

##### 3.2.3.1. Patient’s narrative

*There is some kind of image in my head, that, after all, it’s her profession and she listens to me because that’s what she should do, but beyond her professional interest there is nothing more. It’s a functional relationship. But then…I felt in her response that maybe she doesn’t want it to be so. I mean, I’m not saying a relationship beyond therapy, but…that I would see our relationship not only as a functional relationship, and that it is important to her what I think. I think it surprised me. To some extent it’s a kind of reinforcement because it shows that I also have influence on her, not only does she have influence on me.*

***Interviewer: And how did it make you feel, good?***

*Uh…yes, somewhat, but … it seemed to me like a very small thing.*

Rotem’s PACS markers are characterized, like in the former phases, in a *downplaying* pattern: The therapist’s concerns did not really move her: “*It seemed to me like a very small thing*.”

In terms of the therapeutic distance, the therapist is still *too distant*: She was only doing her job. According to Rotem’s perception, the therapist was not supposed to care. Yet, in this late phase, she understood that the therapist did care, and was quite surprised about it. This understanding encouraged her cooperation, which raised her level of engagement. Yet, on the alliance self-report measure her score was lower than earlier.

##### 3.2.3.2. Therapist’s narrative

The therapist seemed a bit more optimistic at this phase:

*When she started therapy, she couldn’t talk about herself at all. Today we have much more space—she’s able to bring herself and be authentic … But there is also a question I always ask: When there is something so hurt inside of her, how do you reach it? Now I feel I have something to hold on to. But there is also something so impassable, blocked.*

The therapist still feels that Rotem is distant, but also acknowledges some minor new engagement in their relationship.

In summary, the therapeutic relationship was perceived as distant by both partners along the three interviews. The distance was caused, among other reasons, by Rotem’s difficulty in facing and acknowledging emotions and by her tendency to downplay them. The downplaying pattern also resulted in relatively low ratings in the TDS-O scales, at least in the early and mid phases. The patient seemed to hold the distance and prevent possible closeness, but she became embarrassed at the late phase, when she understood that the therapist probably did not want it to remain that way. Although the therapist recognized Rotem’s part in keeping the distance between them, she felt guilty and perceived the therapy as stuck and frustrating. Note that the therapy failed to decrease the patient’s symptoms.

### 3.3. Preoccupied patient: Noam

**Figure 3:**
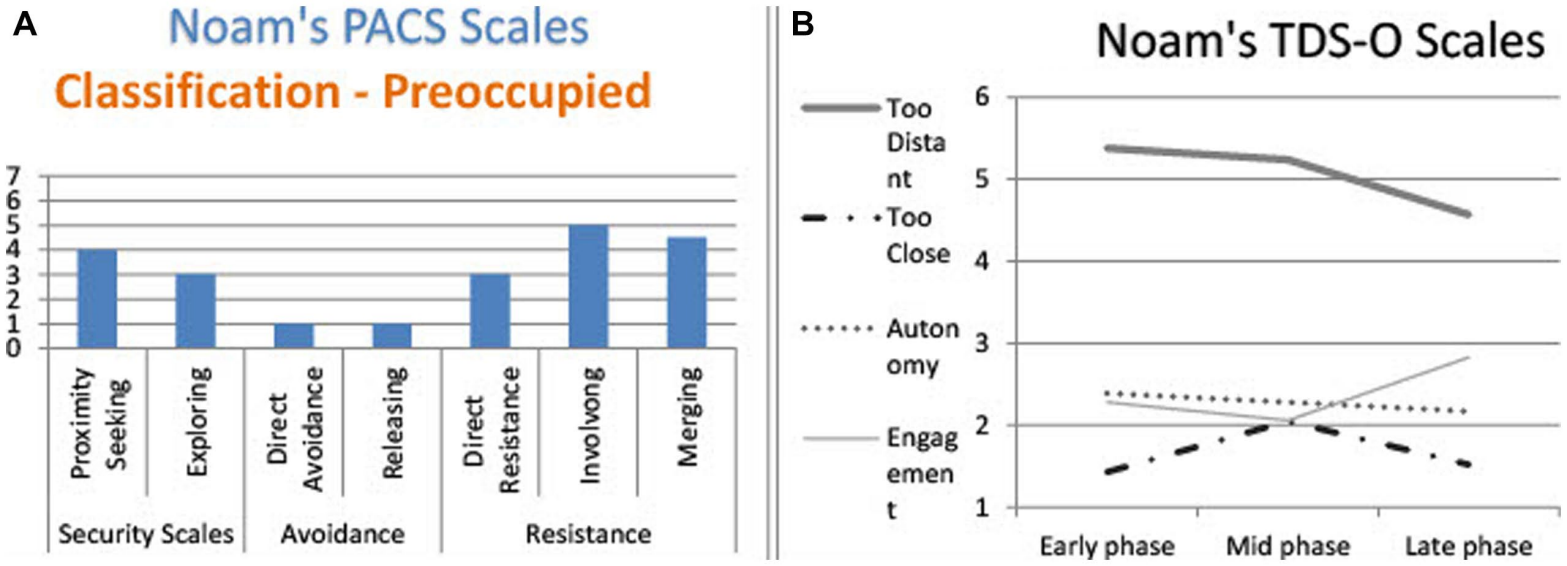
Noam’s TDS-O and PACS markers. **(A)** Noam’s PACS scales. **(B)** Noam’s TDS-O scales.

Noam is a 26-year-old male, single, undergraduate student. His communication patterns were preoccupied according to the PACS (Figure 3A): He employed a lot of proximity seeking*—*describing and elaborating distressing experiences or self-states that aimed to encourage attunement from the listener. This marker is also used by secure patients, but in the preoccupied discourse it is usually accompanied by resistance markers (involving/merging)*—*an implicit attempt to force a listener to accept the point of view of the speaker, and that alone. By doing so, the preoccupied person overrides the listener’s response. This combination of proximity seeking, and resistance is experienced as confusing and overwhelming, and could decrease the attunement of the listener, who may feel unnecessary.

Noam’s TDS-O graph shows that he felt the therapist was *too distant* along the three interviews, together with a slight increase in his engagement, yet no change in his patient’s autonomy (Figure 3B).

In the therapist’s narratives, Noam was perceived as too distant all along, but, surprisingly, reported a relatively high level of engagement, which even rose slightly in the late phase.

*Working Alliance and OQ self-report measures:* Noam’s working alliance scores were low at the early and mid phases (3.81 and 3.86 respectively), lower than among the secure and avoidant patients. At the late phase his alliance increased to 4.64, which was higher than that of the avoidant patient. His OQ scores at intake were extremely high (117), but decreased dramatically after he began therapy (70), remained around that point at mid therapy (74), and decreased more at late therapy (64) to the point of reaching the cut-off point at follow-up (63).

#### 3.3.1. Early phase (after session 5)

##### 3.3.1.1. Patient’s narrative

*I said that I felt uncomfortable in therapy, that I couldn’t connect to her [the therapist] and that she looked too young. She said, “I’m not young,” and I said, “It doesn’t matter, you look young. You don’t take part, it’s only me talking, I could just as well talk with a doll … I don’t remember you or any reactions from you … so what’s the point?” And then she asked me what I wanted. Do I want more interaction? And I like, you tell me. You’re the therapist … She tried to apply things she had learned, and I could not connect. I thought it was problematic.*

This quotation is part of a long monologue in which Noam expressed his frustration with the therapy with quotes from his conversations with the therapist. According to the **PACS,** the narrative is marked as an *involving dialogue*: The patient was frustrated and criticized the therapist in a one-sided description. There was no option available for a listener to elaborate or contribute in a way that could ease Noam’s pain. The possible responses were either identification or distancing.

In terms of **therapeutic distance,** the therapist was perceived as *too distant*: she did not help and seemed cold and distant “*like a doll*.” In addition, he discarded the therapist’s attempts to create engagement: When the therapist asked what he would like, he referred to it as a “protocol” response and rejected it: “*You tell me. You’re the therapist*.”

##### 3.3.1.2. Therapist’s narrative

*I had a good feeling and anticipation during our first session: Yes, here we start. And then suddenly, when this crisis came [referring to the patient’s criticism], I suddenly felt that from his side it’s a big “no” … and I had the feeling that it’s directed at me: like, “Something doesn’t feel good here for me and I wouldn’t like to go on with you.”*

The therapist’s narrative revealed frustration and confusion. She could not figure out what Noam wanted. While she had a sense of a convenient therapeutic distance, she realized that Noam felt distant and maybe even wanted to terminate.

It is interesting, however, that despite the frustration expressed in the interview and the low WAI score, Noam’s symptoms decreased significantly from intake to this early phase, and continued to decrease throughout therapy.

#### 3.3.2. Mid phase (after session 15)

Noam continued to express his frustration and doubt about the effectiveness of the therapy, as well as his criticism of the therapist. The therapist expressed a feeling of coming to a *“dead end.”* She felt his frustration but could not find the right way to help.

#### 3.3.3. Late phase (after session 28)

##### 3.3.3.1. Patient’s narrative

*And then she mentioned that my dream about an intimate relationship is always related to my little brother. As if the love I want to give and feel is like the love I have for him. This is a love that, if I can imagine how I would feel towards my own child, this is like I feel towards him: to give to him, to smile when he smiles, not to think twice before I give something: time, money, gifts, everything … this is totally unconditional.*

***Interviewer: And how did you react to her saying this?***

*That sounds kind of sick … She kind of phrased it like … it sounded bad. But I understand what she meant. I always have dreams—if not about my brother, then I dream about a baby. So, this link kind of explained it.*

***Interviewer: What do you hope for when telling her the dream?***

*I don’t hope too much … I don’t believe … It’s as if I didn’t learn what to expect from her … Sometimes there are connections here and there…the most meaningful sessions are those when talking about the therapy itself. As if, if it’s this or that, and then talking, and we reach a different, unrelated topic, and then there is some improvement or something.*

Noam’s discourse pattern is defined as *direct resistance* in terms of the PACS: He did not answer the interviewer’s question about his hopes (*“I do not hope too much … I do not believe.”*) Another marker is *merging:* He changed his evaluation of the situation from positive to negative. His feelings in the narrative were confusing and unclear, which would again decrease a listener’s ability to take an active part in the conversation.

In terms of the therapeutic distance, Noam perceived the therapist as a bit less distant than during the previous phases, but still did not trust her interpretation, even when it was intended to empower him by showing him how devoted he could be to a person he loves (granting *autonomy*). Noam was not able to absorb this empowerment. At first, he was afraid that she had criticized him for being over-involved with his brother (“*That sounds kind of sick*”), and when he understood what she meant, he became preoccupied with the instability of their ability to connect. Yet, alliance scores increased at this phase and his symptoms continued to decrease.

##### 3.3.3.2. Therapist’s narratives

The therapist, at this phase, discussed issues related to the therapeutic work, and it seems that there is more engagement between them. For example, she related that Noam again raised the question of the purpose of therapy and wondered whether it was helpful. For the first time, she said, she was able to connect his questions to feelings he had outside the room in interactions with other people. She felt that Noam accepted the connection and this helped them touch on some of the main issues of the therapy. Despite this, the therapist still had the feeling that she was unable to help him.

*At a certain moment, after responding to a question he raised, I asked: “Did it help you, to discuss this with me? I’m not sure.” And he answered: “OK, there is still time to decide.” I felt throughout the whole situation as if he was examining me.*

In summary, along the three interviews, the relationship between Noam and the therapist failed to reach a comfortable therapeutic distance. Even moments of engagement were perceived as temporary and fragile. It seems that Noam’s communication patterns, characterized by proximity seeking (through the expression of negative feelings) on the one hand, and the exclusion of the listener through the *merging* and *involving* patterns, on the other, made interaction with him confusing and frustrating. This appears to have negatively influenced the therapist’s ability to attune to him. However, there was a gap between the negative picture depicted in the narratives and self-report of the alliance and the symptom level that are more optimistic.

## 4. Discussion

The purpose of this study was to explore the various pathways that patients with different attachment patterns follow in negotiating therapeutic distance. We used two attachment-informed observational measures: The Patient Attachment Coding System (PACS) and the Therapeutic Distance Scale–Observer version (TDS-O). The results were compatible with the view that the PACS relates to the stable (trait-like) component of patient’s attachment classification, while the TDS-O relates to a more flexible, dynamic (state-like) component—the closeness–distance dyadic “dance.”

This preliminary study shows that the PACS can be implemented on RAP narratives and yield agreement between judges on attachment classifications, even though it was originally designed to be applied to transcripts of therapy sessions. This reinforces the findings of the developers of the PACS, according to which discourse patterns are a universal personality element that can be identified in various types of interaction (40). In addition, analysis of the PACS scales suggested that the discourse patterns remained stable along therapy. Thus, the secure patient used *exploring* and *proximity seeking* at all three phases; the avoidant patient used *direct avoidance* and *releasing;* and the preoccupied patient continued to use *proximity seeking* together with *involving* and *merging* along the three phases.

In contrast, although the discourse patterns seemed stable, the therapeutic distance on the TDS-O scales changed along the three phases. In the case of Doron, the secure patient, he moved from issues of negotiating closeness and distance in the early phase; only after establishing comfortable engagement was he able to focus on issues of autonomy at the mid phase. At the late phase, he re-examined proximity and engagement, and presented a complex picture of these dimensions: While he felt close and secure in his relationship with the therapist, he wondered about the amount of exploration possible when the relationship was so relaxed and comfortable. Rotem, the avoidant patient, experienced the therapist as *too distant* throughout the three interviews, but her *engagement* developed, allowing her to feel more empathy towards the therapist’s attempts, and for the therapist to connect to the patient’s emotional world, which she experienced as “flat.” Finally, Noam, the preoccupied patient, experienced the therapist as *too distant* and unhelpful along the three phases. The therapist was also frustrated at not being able to help him. At the mid and late phases, signs of autonomy and engagement appear in their narratives, but they failed to decrease Noam’s sense of distance and his ambivalence regarding the relationship.

The relative stability throughout the RAP interviews of the PACS discourse patterns on the one hand, and changes in the dimensions of the therapeutic distance, on the other, support the premise that attachment has trait and state components (59). The expansion of the IWM in therapy concerns, according to this preliminary study, aspects of the situational (state), while characteristic attachment as expressed in the discourse patterns remained relatively resistant to change, at least in the 28 sessions of this study.

Exploration of the intersection of the two measures allows us to expand our understanding of therapeutic distance and its expressions in patients with different attachment patterns, shedding light on the therapeutic relationship from an attachment-informed perspective in several respects:

First, the prototype example of avoidant patients implies that the releasing pattern may result in a low to medium rating of the *too distant* and *too close* scales, since even when the patients expressed these feelings, they tended to downplay their statements or attribute the difficulty to external circumstances (“*That’s how it is in therapy”*), and the emotional experience that emerged from the description was minimal. This makes it difficult to identify feelings using narrative-based observational measures like the TDS-O and may obscure the differences between avoidant and secure patients regarding the sense of excessive closeness or engagement.

Second, the prototype example of the preoccupied patient implies that such patients tend to intensify the emotional experience and thus receive high ratings on the TDS-O proximity–distance scales (with an emphasis on *too distant*), but their speech patterns are full of details or vague speech, leaving the listener out of the conversation. This should be especially noted when assessing the *autonomy* and *engagement* scales. It is possible that these patients will dismiss the therapist’s efforts to grant autonomy or to encourage engagement; however, the case example shows that there may be a gap between the observer’s impression and the preoccupied patient’s self-reports, which might be more positive than the observer’s regarding both the alliance and the symptom level. This gap may be related to preoccupied patients’ need to keep the listener close and avoid possible abandonment by intensifying their distress.

In addition, contrary to the original therapeutic distance model (23), the RAP interviews show that secure patients may also experience the therapist as more distant or close than they would like at the moment. This corresponds with the findings of Miller-Bottome et al. (42), who showed that secure patients can also have trouble regarding closeness. However, their ability to express feelings freely and reflect on them, as well as the initiative they might take to change the uncomfortable situation, allow the therapist to get closer to their emotional experience and respond to it with a wide variety of reactions. Similiarly, Miller-Bottome et al. (43), studying alliance rupture resolution with the PACS, demonstrated, using examples from session transcripts, that “secure patients are particularly responsive to resolution strategies that focus on the here-and-now, while insecure patients’ (avoidant and preoccupied) characteristic ways of communicating pose significant challenges to the resolution process” (p. 175).

Finally, the narrative-based observation may point to some differences in the way engagement is perceived and negotiated by different patients. The secure patient took the initiative of engaging with the therapist; the preoccupied patient placed responsibility for engaging on the therapist, but unconsciously prevented the possibility of engaging through his confusing and vague communication, especially when asked about his feelings or wishes; the avoidant patient tried to dismiss the need for engagement and underestimated its importance. In addition, the analysis of the patients’ as well as the therapists’ relational narratives extends previous findings showing a relationship between patients’ ability to explore and a secure attachment to the therapist (60). The narratives demonstrated the possible influence of the ability to explore on the therapist’s interventions: Doron’s therapist noted their ability to work through ruptures through inquiry, while both Rotem and Noam’s therapists remained very cautious in their interventions.

As mentioned, this is a preliminary study, and as such has a number of limitations. First, we only assessed three single cases, so the results must be taken with caution. In this context, it is important to note that although the patients suffered from depression and anxiety, they were functioning young adults and may not reflect more severe patients. In addition, the use of prototypical patients for this demonstration raises a question about less prototypical patients’ communication patterns and their closeness–distance dynamics. A study of a larger sample needs to be conducted, including relating the TDS-O measure with outcome, which emphasizes the need for caution in drawing conclusions.

Second, we only assessed patients’ attachment discourse. It is presumed that therapist communication patterns would also have an impact on the closeness–distance dynamics in the dyad (61–64). In addition, it is important to note that patients and therapists of each dyad were interviewed separately in RAP interviews. We did not assess their real in-session transcripts, therefore, our understanding of patients’ influence on therapist attunement (64) is only estimated; it is based on the patients’ responses to the interview, as well as therapist narratives (which were assessed at the same time points and emotional parameters of closeness and distance as the patients’). Nevertheless, while during the therapeutic session therapists intervene relying on their professionalism and experience in attuning to patients’ needs, the narratives in the RAP interview encourage expression of their feelings that are not usually apparent to the patient.

To the best of our knowledge, this is the first study to apply the PACS to analyzing narratives collected through RAP interviews. Our preliminary findings support the use of the PACS for identifying attachment patterns in different kinds of conversation-based transcripts related to mental states (19, 40), and therefore expand the application of the PACS as an effective attachment-based assessment in a variety of observational studies.

Although time-consuming and expensive to implement, observing the therapeutic relationship with the PACS and the TDS-O allows expansion of understanding the in-session attachment dynamics with different patients. An example is the understanding that an “ideal” therapeutic distance is not a goal to be achieved at early phase of therapy, but a dynamic process that may change throughout therapy. At the late phase, a feeling of distance may re-emerge, possibly related to the approaching termination, and may raise automatic defense mechanisms relating to patient attachment. In addition, the inquiry reveals additional more implicit aspects of the alliance that cannot be identified through traditional self-report measures.

We believe that this article has implications for therapist training and supervision, as it suggests different pathways through which patients’ attachment classifications may affect the therapeutic relationship. For example, the avoidant patients’ tendency to downplay emotions may be reflected in therapists’ feelings of boredom, guilt, and self-doubt. Preoccupied patients can be confusing and overwhelming because of their tendency to combine proximity seeking and resistance. It has been suggested that the consequences of this kind of combination indeed leads therapists to feel irritated and overwhelmed (65, 66). Therapists’ familiarity with research on attachment classifications in sessions and the ways patients with different attachment patterns may express their wishes for proximity could improve their ability to attune to their patients’ emotional needs. Further research is needed to examine possible therapist responses that would allow working with these discourse behaviors to facilitate appropriate responsiveness and collaboration that would enable a corrective emotional experience.

## Data availability statement

The raw data supporting the conclusions of this article will be made available by the authors, without undue reservation.

## Ethics statement

The studies involving human participants were reviewed and approved by Ethics Committee of the Paul Baerwald School of Social Work and Social Welfare at the Hebrew University. The patients/participants provided their written informed consent to participate in this study.

## Author contributions

SE, HW, and AT contributed to conception and design of the study. AT trained SE and HW for the observation using the PACS and coded the interviews, together with SE. SE organized the database, performed the statistical analysis and wrote the first draft of the manuscript, with the help of HW. All authors contributed to the article and approved the submitted version.

## Conflict of interest

The authors declare that the research was conducted in the absence of any commercial or financial relationships that could be construed as a potential conflict of interest.

## Publisher’s note

All claims expressed in this article are solely those of the authors and do not necessarily represent those of their affiliated organizations, or those of the publisher, the editors and the reviewers. Any product that may be evaluated in this article, or claim that may be made by its manufacturer, is not guaranteed or endorsed by the publisher.
